# Supplementary material for: Parental Anxiety, Practices, and Parent–Child Relationships among Families with Young Children in China: A Cross-Sectional Study
Source: Children (Basel). 2023 Aug 15;10(8):1388. doi: 10.3390/children10081388 (PMC10453797; doi:10.3390/children10081388)
Supplement: Supplementary file 1 [file children-10-01388-s001.zip › children-2510648-supplementary.pdf]

Table S1. Univariate analysis of factors influencing parental anxiety, practices, and parent-child relationships.

Characteristic	Level of Parenting Anxiety		Mother's Perceptions of Warmth Towards Her Child		Mother's Perceptions of Invasiveness towards Her Child		Change in Frequency of Reading Books or Looking at Picture Books with Child		Change in Frequency of Telling Stories to Child	
	**Statistic**	***p* Value**	**Statistic**	***p* Value**	**Statistic**	***p* Value**	**Statistic**	***p* Value**	**Statistic**	***p* Value**
Caregiver's role	6.0	0.109	NA	NA	NA	NA	0.5	0.912	0.8	0.852
Caregiver's sex	5569.0	0.148	340.5	0.052	585.0	0.532	6329.0	0.789	6301.5	0.753
Caregiver's age	3.6	0.313	2.8	0.246	1.8	0.404	7.2	0.066	8.1	0.043 *
Caregiver's educational level	5.9	0.205	3.0	0.561	8.4	0.078	4.9	0.301	5.4	0.244
Caregiver's occupation	19.6	0.051	6.5	0.837	6.8	0.818	33.3	<0.001 *	31.6	0.001 *
Caregiver's marital status	2.3	0.320	0.3	0.573	0.1	0.719	0.2	0.924	0.2	0.897
Annual household income (CNY)	2.6	0.860	2.9	0.824	4.3	0.634	5.7	0.453	3.9	0.684
Family residence (district)	7.0	0.730	9.5	0.485	8.9	0.538	25.0	0.005 *	25.9	0.004 *
Whether child has siblings	23080.0	0.609	20209.5	0.485	20098.5	0.270	21753.5	0.105	22516.0	0.321
Child's age	15.6	0.048 *	10.6	0.227	3.6	0.888	15.9	0.043 *	12.9	0.116
Whether the child was born prematurely	5439.0	0.486	4582.0	0.562	4499.5	0.302	5261.0	0.311	5613.0	0.670
Whether the child was born with low birth weight	3993.0	0.148	4165.5	0.520	3835.5	0.056	4676.0	0.835	4326.0	0.384
Whether the child was admitted to the NICU	14598.5	0.260	12838.0	0.586	12607.0	0.247	14541.5	0.225	14573.5	0.233
Feeding pattern of the child	8.6	0.014 *	2.9	0.238	1.2	0.546	0.1	0.959	1.3	0.532
Number of family members living together during lockdown	4.6	0.602	5.4	0.499	9.5	0.145	5.6	0.475	6. 5	0.372
Whether someone you know was infected by COVID-19 during lockdown	18978.5	0.005 *	18332.5	0.312	18707.5	0.426	22168.5	0.885	22044.0	0.799
Whether working from home during lockdown	22380.0	0.149	21032.5	0.860	20440.0	0.275	20322.5	0.001 *	19536.0	<0.001 *
Whether facing parenting issue of child feeding	12114.5	<0.001 *	11976.5	0.350	12379.0	0.670	13916.0	0.363	14529.5	0.879
Whether facing parenting issue of child physical development	12659.5	0.001 *	12210.0	0.582	12320.0	0.607	13027.5	0.031 *	12861.5	0.017 *
Whether facing parenting issue of child other development	13491.0	0.026 *	12230.0	0.504	12571.0	0.846	14447.0	0.546	14690.0	0.757
Whether facing parenting issue of parent-child interaction	13396.0	0.040 *	11706.0	0.226	11824.0	0.165	14033.5	0.396	14084.5	0.427
Whether facing other parenting issues	3637.5	0.812	3402.5	0.996	3246.0	0.528	3263.5	0.269	3587.5	0.753
Whether facing external parenting difficulty in accessing professional guidance from childcare providers in CHCs	12986.5	0.004 *	11831.0	0.201	12419.0	0.610	14248.0	0.459	13689.5	0.150
Whether facing external parenting difficulty in accessing professional guidance from pediatricians in hospitals	12538.5	<0.001 *	12583.5	0.834	12437.0	0.558	14547.5	0.611	14439.5	0.517
Whether facing external parenting difficulty in fear of child being infected	13254.5	0.964	11272.5	0.608	11169.0	0.359	12817.5	0.546	12766.5	0.499
Whether facing other external parenting difficulties	5454.0	0.751	5043.0	0.902	4836.0	0.399	5528.0	0.917	5091.5	0.323

* Indicates statistically significant results ($p < 0.05$).
